# Galloflavin Relieves the Malignant Behavior of Colorectal Cancer Cells in the Inflammatory Tumor Microenvironment

**DOI:** 10.3389/fphar.2021.752118

**Published:** 2021-12-10

**Authors:** Li Guo, Yi Yang, Yongjia Sheng, Jin Wang, Wenyan Li, Xiaohong Zhou, Shuiliang Ruan, Chenyang Han

**Affiliations:** ^1^ Department of Center Laboratory, The Second Affiliated Hospital of Jiaxing University, Jiaxing, China; ^2^ Department of Pharmacy, The Second Affiliated Hospital of Jiaxing University, Jiaxing, China

**Keywords:** galloflavin, inflammatory microenvironment, NLRP3, colorectal cancer, malignant behavior

## Abstract

**Background:** In this study, we mainly aimed to explore the correlation between galloflavin and NLRP3 and its effect on colorectal cancer.

**Methods:** NLRP3 was overexpressed in SW480 cells; LPS + ATP was used to mimic the inflammatory microenvironment. Wound healing assay and Transwell assay were utilized to detect cell migration and invasion abilities; CCK-8 assay was performed to detect cell viability alterations; colony formation assay was conducted to detect colony formation ability; Western blot was used to detect the levels of NLRP3, ASC, C-Myc, and P21. SW480 cells were pretreated with high-dose and low-dose galloflavin, followed by observation of their effects on cell metastasis and invasion. NLRP3 was knocked out in SW480 to construct the SW480-NLRP3^−/−^ cell line, followed by high-dose galloflavin treatment and subsequent observation of cell metastasis and invasion abilities. Small molecule–protein docking and pull-down assay were performed to confirm the targeting relationship between galloflavin and NLRP3. After constructing a tumor-bearing mice model, galloflavin was intragastrically administered, followed by detection of tumor growth, expression of NLRP3 and ASC by immunohistochemistry, and tumor histopathology by H&E staining.

**Results:** After NLRP3 overexpression and LPS/ATP induction in SW480, the cell migration and invasion abilities were significantly enhanced, and cell viability was also enhanced. The activation of NLRP3 could promote the malignant behavior of colorectal cancer cells in the inflammatory microenvironment. Galloflavin treatment could significantly attenuate the malignant behavior of SW480 in the inflammatory microenvironment and inhibit the migration and invasion capabilities of SW480. The knockout of NLRP3 inhibited the effect of galloflavin, which did not significantly change the migration and invasion abilities. Molecular docking and pull-down assay revealed a targeted binding relationship between galloflavin and NLRP3 and that galloflavin is bound to NLRP3 not ASC protein. Moreover, galloflavin could inhibit tumor growth and decrease the expression of NLRP in tumor-bearing mice.

**Conclusion:** In this study, we found that NLRP3 could promote the migration and invasion of colorectal cancer cells in the inflammatory microenvironment. Galloflavin could inhibit the malignant behavior of colorectal cancer cells by targeting NLRP3.

## Background

Various reasons can induce the occurrence of inflammatory states in the tumor microenvironment (TME) (Ural et al., 2021). In turn, the inflammatory microenvironment can promote tumor growth, invasion, and metastasis by mediating complex pathways (Rada et al., 2020). Inflammatory TME is defined as the inflammatory internal environment during tumorigenesis and tumor progression, which is mainly composed of interstitial fibroblasts, blood vessels and lymphatic network, extracellular matrix components, massive inflammatory cells, and inflammatory factors (Bauer et al., 2020). This special “biological system” involves the mutual regulation and influence of various cells, cytokines, and chemical factors, rendering the complicated and dynamic inflammatory TME (He et al., 2021; Zhou et al., 2021). Inflammasomes play an important role in the occurrence and development of inflammation-associated diseases. Among them, NLRP3 inflammasomes can be activated by various pathogen-associated molecular patterns (PAMPs) and damage-associated molecular patterns (DAMPs) (Coll et al., 2015). It further activates caspase-1, releases mature forms of Interleukin-1*β* (IL-1β) and Interleukin-18 (IL-18), causes inflammatory response in the body, and participates in the occurrence and development of many diseases, including type 2 diabetes, gout, atherosclerosis, neurodegenerative diseases, tumor, and inflammatory bowel disease. (Jiang et al., 2017; Perera et al., 2018). At present, NLRP3 has been revealed to be associated with the progression of non–small cell lung cancer, osteosarcoma, and other malignant tumors, which is related to the activation of NF-κB-STAT1/3 (Pellegrini et al., 2018). Therefore, NLRP3 is expected as a novel target for tumor and microenvironment regulation [10].

Galloflavin (Gal), an LDH-A/B inhibitor (Guo et al., 2014; Wendt et al., 2020), has been found to exert a certain role in inflammatory regulation. Therefore, in this study, we used NLRP3 as the starting point to investigate the target of Gal and its anti-colorectal cancer effect.

## Materials and Methods

### Materials and Reagents

Colorectal cancer cell line SW480 (Procell, Wuhan, China), galloflavin (Topscience, Shanghai, China, 98% purity), lipopolysaccharide (LPS), and adenosine triphosphate (ATP) (Sigma, United Sates), Cell Counting Kit-8 (CCK-8) (MCE, Shanghai, China), monoclonal antibodies against NLRP3, ASC, C-Myc, and P21 (Abcam, United Sates), NLRP3 overexpression plasmid pCDH-NLRP3 (Invitrogen, United Sates), BCA protein quantitation kit (Beyotime Biotechnology Company, Shanghai, China), DAB immunohistochemical staining kit (Abcam, United Sates), Hematoxylin and Eosin (H&E) staining kit (Beyotime Biotechnology, Shanghai, China), DMEM high glucose medium (Gibco, United Sates) and ELISA kits of IL-1β, IL-18, and TNF-α (Nanjing Jiancheng Biotechnology Co., Ltd., Nanjing, China) were purchased. Balb/c nude mice were maintained at Jiaxing University.

### SW480 Cell Culture and Gal Intervention [13]

SW480 cells were cultured in DMEM containing 10% fetal bovine serum (FBS) at 37°C with 5% CO_2_. SW480 cells were passaged after 3–5 days, followed by cell viability detection using trypan blue. Cells were divided into different groups: the DMSO group was the control cell; cells in the L/A group were treated with lipopolysaccharide (LPS) (0.1 g/L), and adenosine triphosphate (ATP) (1 μM) mimics the inflammatory microenvironment; and cells in the Gal groups were pretreated with 5 and 15 μM Gal for 6 h, followed by L/A intervention for 6 h.

### Detection of Cell Viability (CCK-8)

SW480 cells of the logarithmic phase were inoculated into 96-well plates and cultured overnight. After cell adherence, cells were pretreated with Gal and further treated with LPS and ATP. The cell viability was measured at 6, 12, 24, and 48 h. In brief, 10 μL of the CCK-8 reagent was added to each well for 4 h, and fresh serum-free medium was replaced (100μL/well). The absorbance was measured at 450 nm by using a microplate reader. The absorbance of blank medium was removed, and the absorbance at 0 h was used as the control to calculate cell viability. Results were shown as %, and three replicates were set in each group.

### Detection of Cell Migration and Invasion Ability (Transwell Chamber)

The cell culture medium was changed to serum-free medium 12 h before the experiment. In brief, 40 μL Matrigel was coated into the Transwell chamber. Cells were digested, washed with 1 μL PBS twice, and 500 μL serum-free medium was added to the 24-well plate. A total of 5×10^5^ cells were resuspended, and 200–250 μL of cell suspension was added to the Transwell chamber to ensure that there were no bubbles between the lower complete medium and the Transwell chamber. After cell adherence, cells were treated with Gal and further treated with LPS + ATP, followed by incubation for 24 h. Afterward, cells were stained with 500 μL of 0.1% crystal violet staining solution (prepared with methanol and diluted with PBS) at room temperature in dark for 15 min, rinsed with PBS, wiped with a cotton swab, and dried. Cells were observed under an inverted fluorescence microscope (200x) to count and photograph the number of cells penetrating through the membrane. Five fields of view were randomly selected for cell counting, followed by calculation of the average value. Matrigel was not added for the migration assay. The number of migrated cells was directly counted, and three replicates were set in each group.

### Wound Healing Assay

SW480 cells were inoculated in the 24-well plate at a density of 5×10^4^ cells/well. After cell adherence overnight, a sterile 10 μL pipette tip was used to scratch a straight line, and 100 μL PBS was subsequently used to discard cell debris, followed by incubation in a serum-free culture medium at 37°C with 5% CO_2_. Cells were treated with Gal and further treated with LPS + ATP. The cell migration and the width of the scratch were observed under an inverted microscope at 0 and 24 h. The image analysis software ImageJ was used to measure the width of the cell scratch. The migration rate was calculated according to the formula: cell migration rate = (cell scratch width_24h_ − cell scratch width_0h_)/cell scratch width_0h_ × 100%, and three replicates were set in each group.

### Colony Formation Assay

Cells were inoculated into a 6-well plate at a density of 5×10^3^ cells/well and further incubated. Fresh culture medium was changed every other week, and cells were subjected to Coomassie blue staining after 2 weeks. A cell clone with over 30 cells was counted as a colony. A cell colony was observed and photographed under a light microscope at 100x magnification. Five fields of view were randomly selected to record the number of colonies, followed by calculation of the average value. Three samples were set in each group, and the experiment was performed three times.

### Western-Blot

Cells were inoculated into 6-well plates at a density of 1×10^6^ cells/mL. Cells were treated with Gal, subsequently treated with LPS + ATP and digested. The collected cells were lysed within 500 μL of the RIPA protein lysate on ice for 30 min and centrifuged at 12,000 r/min at 4°C for 10 min. The supernatant was collected and subjected to protein concentration by using the BCA kit. The protein sample was mixed with the loading buffer and boiled at 100°C for 5 min. The prepared protein sample (25 μL/well) was subjected to SDS-PAGE gel (5% concentration gel and 10% separation gel) at 60 V and subsequently at 120 V, transferred to the membrane at 4°C for 1.5 h. The PVDF membrane was blocked with 5% skimmed milk powder for 2 h, incubated with monoclonal antibody at 4°C overnight, washed with TBST, incubated with HRP-labeled goat anti-rabbit IgG at 37°C for 2 h, and visualized with ECL, followed by image acquisition by an automatic gel imaging system. GAPDH was used as an internal control to analyze the protein expression level.

### Enzyme-Linked Immunosorbent Assay

The cell culture supernatant was collected to determine the levels of IL-6, TNF-α, and IL-1β in cell experiments. In brief, the supernatant was collected and centrifuged at 3000 g, followed by detection of inflammatory factors (including IL-1β, IL-18, and TNF-α) by ELISA kits. The absorbance value was measured at 450 nm by using a microplate reader (BioTek, United Sates) according to the manufacturer’s instructions, and the results were expressed in pg/ml.

For the animal experiment, after the mice were sacrificed by carbon dioxide asphyxiation, the tumor tissue was ground with liquid nitrogen, lysed within 1.0 ml of RIPA lysate on ice for 30 min, and centrifuged at 10000 g for 15 min, followed by protein quantification of the supernatant. The levels of inflammatory factors were measured according to the manufacturer’s instructions.

### Validation of the Targeting Relationship Between Galloflavin and NLRP3

#### Molecule–Protein Docking

The receptor protein NLRP3 was retrieved from the Protein Data Bank (http://www.rcsb.org/pdb) database. PYMOL 2.3.4 software was utilized to remove water and the ligand on the receptor protein, AutoDockTools software was used for modification on the receptor protein, such as hydrogenation and charge balance, and AutoDock Vina 1.1.2 was used for molecular docking as well as binding energy scoring between the receptor protein and the small ligand molecule. The results were output by AutoDock Vina as Affinity. By calculating the steric effect, repulsion, hydrogen bond, hydrophobic interaction, and molecular flexibility of the receptor–ligand complex, a comprehensive score was calculated and used to evaluate its affinity, which was an important indicator to measure whether the ligand could effectively bind to the receptor molecule. It was the core parameter of AutoDock Vina software. The lower the energy value indicated, the better the binding effect of the two.

#### Pull-Down

NLRP3 recombinant protein was combined with biotin-labeled galloflavin (Biotin-Galloflavin). Recombinant protein G magnetic beads were incubated with the NLRP3 antibody. After washing with Tris buffer, NLRP3 was detected by Western blot accordingly, followed by biotin detection using the horseradish peroxidase–conjugated antibiotin antibody (CST, Boston, United Sates).

### The Effect of Galloflavin on Tumor-Bearing Mice

The animal experiment is approved by Jiaxing University ethics approval. Nude mice, 4–5 weeks old, 19–23 g of weight, were raised in the SPF environment. SW480 cells were cultured to the logarithmic phase and digested, followed by the adjustment of cell concentration to 3 × 10^7^/ml. Afterward, 0.2 ml cell suspension (6 × 10^6^ cells) was injected into the right hind limb after disinfection. Nodules appeared at the injection site within 3–4 days. Mice were subsequently divided into control and galloflavin groups. Mice of the galloflavin group were administered with 5 mg/kg and 15 mg/kg galloflavin once daily. Mice were kept for 15 days and sacrificed by carbon dioxide asphyxiation, followed by tumor resection and measurement of tumor volume.

### H&E Staining

The tumor tissues of mice were embedded in paraffin, serially cut into 4 μm-thick sections, deparaffinized with xylene, dehydrated with gradient ethanol, rinsed with tap water for 2 min, stained with hematoxylin for 3 min, rinsed with tap water for 2 min, treated with 1% acid alcohol for 2 s, rinsed with tap water for 2 min, treated with 1% ammonia water for 20 s, treated with 0.5% eosin alcohol for 10 s, dehydrated with gradient alcohol, transparent with xylene, and sealed with neutral gum. Finally, the pathological change of the liver tissue was observed under a light microscope.

### Immunohistochemistry Staining

Tumor tissue sections were deparaffinized in xylene three times, soaked in absolute ethanol for 5 min, and immersed in 95–85% ethanol twice. The slices were immersed in 0.01 mol/L citrate buffer (PH = 6.0), subjected to antigen retrieval using microwave at 98°C for 20 min, cooled down at room temperature for 30 min, and rinsed with distilled water. The slices were incubated within 3% hydrogen peroxide at room temperature for 10 min to eliminate endogenous peroxidase, blocked with 2% BSA, incubated with the monoclonal antibody (dilution 1:300) at 4°C, incubated with peroxidase-labeled streptomycin for 15 min, rinsed with PBS three times (5 min each time), and visualized with freshly prepared DAB. After observing the visualization reaction under a microscope, the slices were counterstained with hematoxylin and mounted, followed by observing under an upright Olympus-BX51 microscope.

## Statistical Analysis

SPSS 20.0 software was used for statistical analysis. Measurement data were expressed as mean ± standard deviation (±s). One-way ANOVA was used for comparison between multiple groups, and the SNK test was used for comparison between groups. *p* < 0.05 indicated statistical significance.

## Results

### NLRP3 Could Promote the Malignant Behavior of SW480 Cells in the Inflammatory Environment

**FIGURE 1:**
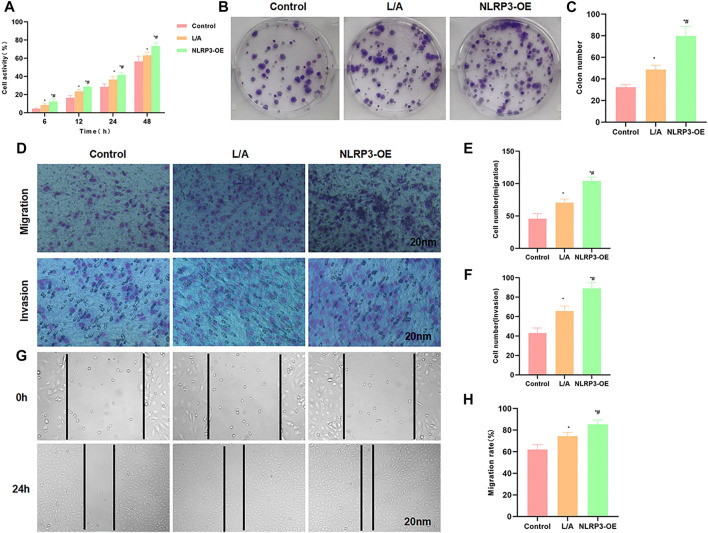
NLRP3 promoted the malignant behavior of SW480 in the inflammatory microenvironment. **(A)**: The detection of cell viability revealed that cell viability was significantly upregulated in the inflammatory microenvironment. NLRP3 overexpression could further promote cell viability. Comparison with the control group, ^*^ *p* < 0.05; comparison with the L/A group, ^#^ *p* < 0.05. **(B,C)**: Colony formation assay showed that LPS/ATP induced colony formation in SW480, with more number of colonies than that of control. Overexpression of NLRP3 could further promote colony formation. Statistical analysis of the number of colonies, comparison with the control group, ^*^ *p* < 0.05; Comparison with the L/A group, ^#^ *p* < 0.05. **(D–F)**: Detection of cell invasion and migration by Transwell assay revealed that NLRP3 could promote the migration and invasion ability of SW480 in the inflammatory microenvironment. Comparison with the control group, ^*^ *p* < 0.05; comparison with the L/A group, ^#^ *p* < 0.05. **(G,H)**: Wound healing assay showed that NLRP3 could promote the migration of SW480 cells. Analysis of the migration rate, comparison with the Control group, ^*^ *p* < 0.05; comparison with the L/A group, ^#^ *p* < 0.05.

We performed overexpression of NLRP3 in SW480 cells, followed by LPS and ATP treatment to induce the inflammatory microenvironment. As a result, L/A induction could promote the viability and promote cell proliferation of SW480 cells; the overexpression of NLRP3 (NLRP3-OE) further upregulated the cell viability, which was significantly higher than that of L/A (Figure 1A). Colony formation assay also showed that the number of colonies of L/A was significantly higher than that of control. NLRP3-OE could further promote colony formation in the inflammatory environment, with a significantly higher number of colonies than that of L/A (Figures 1B,C). Cell migration and invasion assays also showed that L/A promoted the migration and invasion of SW480 cells, with more cells than that in the control group. NLRP3-OE further upregulated cell migration and invasion abilities, which were significantly higher than those of L/A (Figures 1D–F). Wound healing assay also demonstrated that the migration rate of NLRP3-OE cells was significantly upregulated, which was higher than that of L/A and control (Figures 1G,H).

**FIGURE 2:**
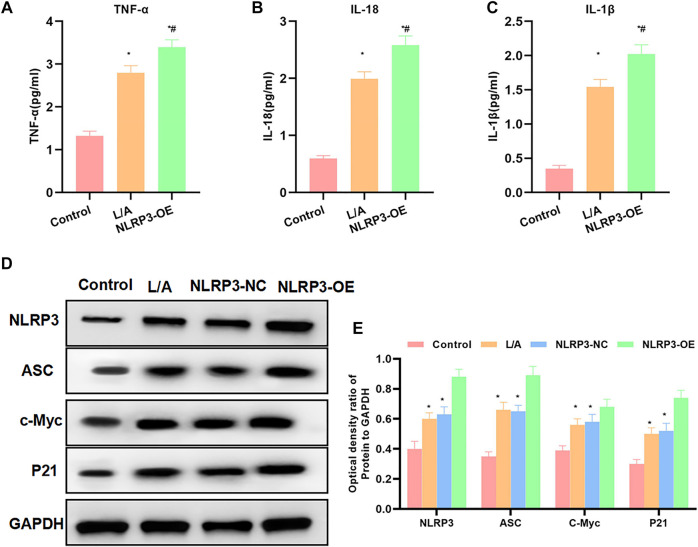
Effect of NLRP3 on inflammatory factors and protein expression levels. **(A–C)**: LPS/ATP could promote the level of inflammatory factors in SW480, with significantly increased levels of IL-6, TNF-α, and IL-1β. The level of inflammatory factors in NLRP3-OE was further upregulated. Comparison with the Control group, ^*^ *p* < 0.05; comparison with the L/A group, ^#^ *p* < 0.05. **(D,E)**: The inflammatory microenvironment could promote the activation of NLRP3 and promote the expression of c-Myc and P21. The expression of c-Myc and P21 was further upregulated in NLRP3-OE. Comparison with the control group, ^*^ *p* < 0.05; comparison with the L/A group Compare, ^#^ *p* < 0.05.

The detection of inflammatory factors found that L/A could promote the expression of inflammatory factors. To be specific, the expression levels of IL-6, TNF-α, and IL-1β were significantly higher than those in the control group. NLRP3-OE could further improve the levels of IL-6, TNF-α, and IL-1β in the inflammation environment, which were higher than those in the L/A group (Figures 2A–C). When detecting the protein expression, we found that LPS/ATP could promote the expression of NLRP3, c-Myc, and P21 and promote cell proliferation. The inflammatory microenvironment could promote the malignant behavior of SW480. The expression of c-Myc and P21 was further upregulated in NLRP3-OE, indicating that NLRP3 could promote the proliferation of SW480 (Figures 2D,E). Taken together, NLRP3 could promote the malignant behavior of SW480 cells in the inflammatory microenvironment.

### Galloflavin Inhibited the Malignant Behavior of SW480 in the Inflammatory Microenvironment

**FIGURE 3:**
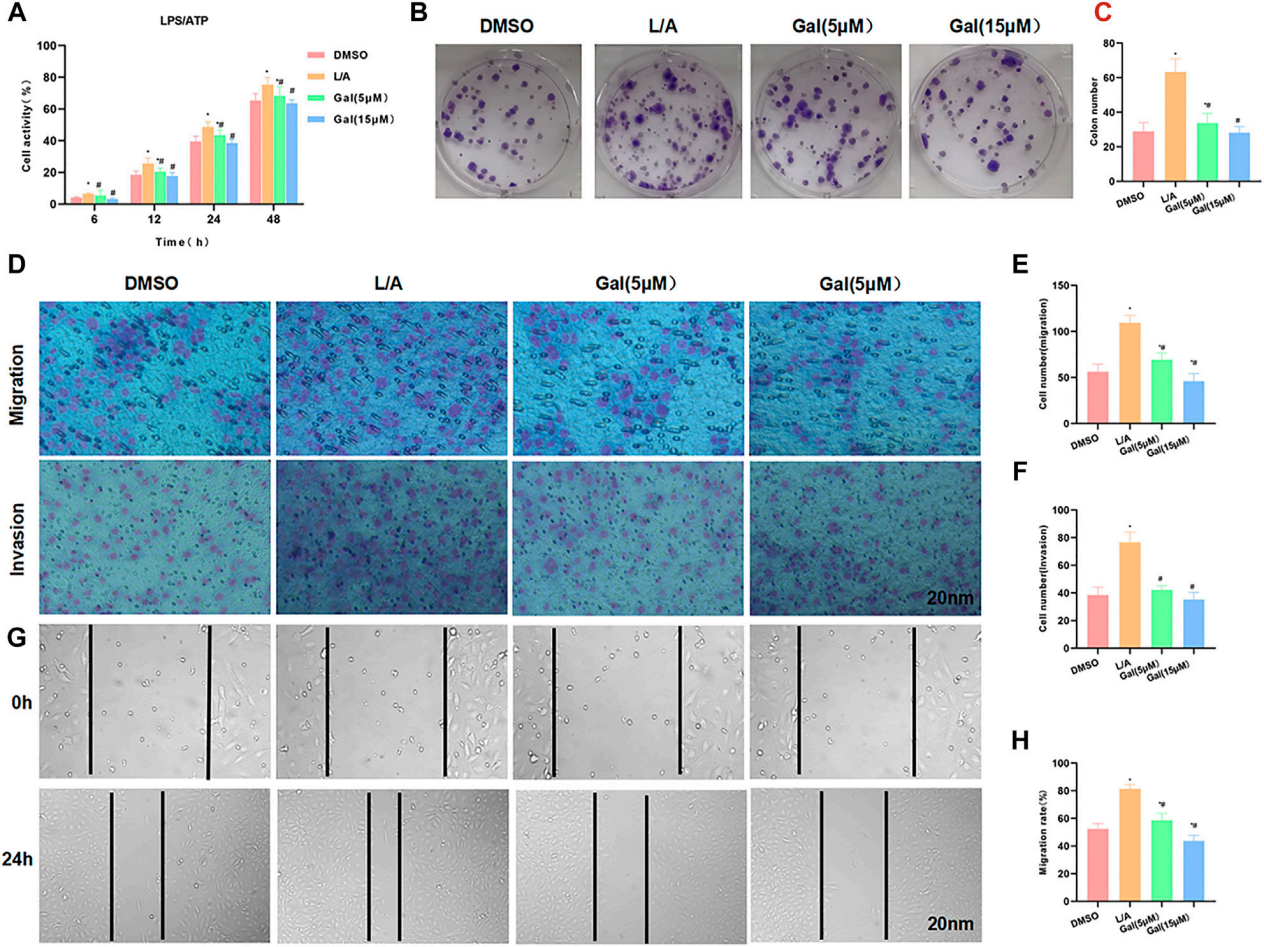
Galloflavin inhibited the malignant behavior of SW480 in the inflammatory microenvironment. **(A)**: Cell viability assay revealed that Gal could inhibit cell proliferation in the inflammatory microenvironment, with a significantly decreased cell viability. High-dose Gal could better inhibit cell viability than that of low-dose. Comparison with the DMSO group, ^*^ *p* < 0.05; Comparison with group A, ^#^ *p* < 0.05. **(B,C)**: Colony formation assay showed that Gal could inhibit the colony formation of SW480, with a significantly lower number of colonies lower than L/A. Comparison with the DMSO group, ^*^ *p* < 0.05; comparison with the L/A group, ^#^ *p* < 0.05. **(D–F)**: Cell migration and invasion assay revealed Gal inhibited the migration and invasion of SW480, with a significantly downregulated cell number. Comparison with the DMSO group, ^*^ *p* < 0.05; comparison with the L/A group, ^#^ *p* < 0.05. **(G,H)**: Wound healing assay showed that Gal inhibited the cell migration rate. Comparison with the Control group, ^*^ *p* < 0.05; comparison with the L/A group, ^#^ *p* < 0.05.

LPS/ATP was used to simulate the inflammatory environment. Gal intervention could inhibit the upregulation of cell viability in the inflammatory microenvironment. Cell viability in Gal was significantly lower than that in the L/A group, with an obvious effect of high-dose Gal than low-dose (Figure 3A). Colony formation showed that Gal could inhibit colony formation of SW480 cells in the inflammatory environment, with a significantly lower number of colonies than L/A (Figures 3B,C). Cell invasion and migration assay showed that Gal inhibited the migration and invasion of SW480 in the inflammatory environment, with a significantly lower number of migrated and invaded cells than that of the L/A group (Figures 3D–F). Wound healing assay also showed that Gal inhibited the migration ability of cells (Figures 3G,H).

**FIGURE 4:**
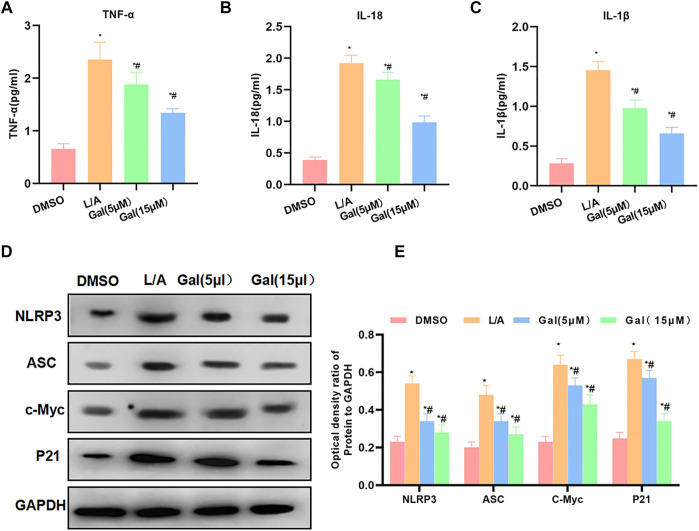
Galloflavin inhibited the expression of inflammatory factors and NLRP3. **(A–C)**: Gal could inhibit the expression of inflammatory factors in the inflammatory microenvironment. The levels of IL-6, TNF-α, and IL-1β of the Gal group were significantly downregulated than that of the L/A group. In addition, high-dose Gal could better inhibit the expression of inflammatory factors than low-dose. Comparison with the DMSO group, ^*^ *p* < 0.05; comparison with the L/A group, ^#^ *p* < 0.05. **(D,E)**: Gal could inhibit the expression of NLRP3, downregulate the levels of NLRP3 and ASC, and suppress the expression of c-Myc and P21. Comparison with the DMSO group, ^*^ *p* < 0.05; comparison with the L/A group, ^#^ *p* < 0.05.

Detection of inflammatory factors showed that Gal could inhibit the release of inflammatory factors. The levels of IL-6, TNF-α, and IL-1β were significantly downregulated, which was lower than that of L/A. High-dose Gal had a higher ability to inhibit inflammatory factors than that of low-dose (Figures 4A–C). Protein detection revealed that Gal could inhibit the expression of P21 and c-Myc. Most importantly, Gal could also inhibit the level of NLRP3 (Figures 4D,E).

### Galloflavin Cannot Further Inhibit the Malignant Behavior of SW480-NLRP3^−/−^Cells

**FIGURE 5:**
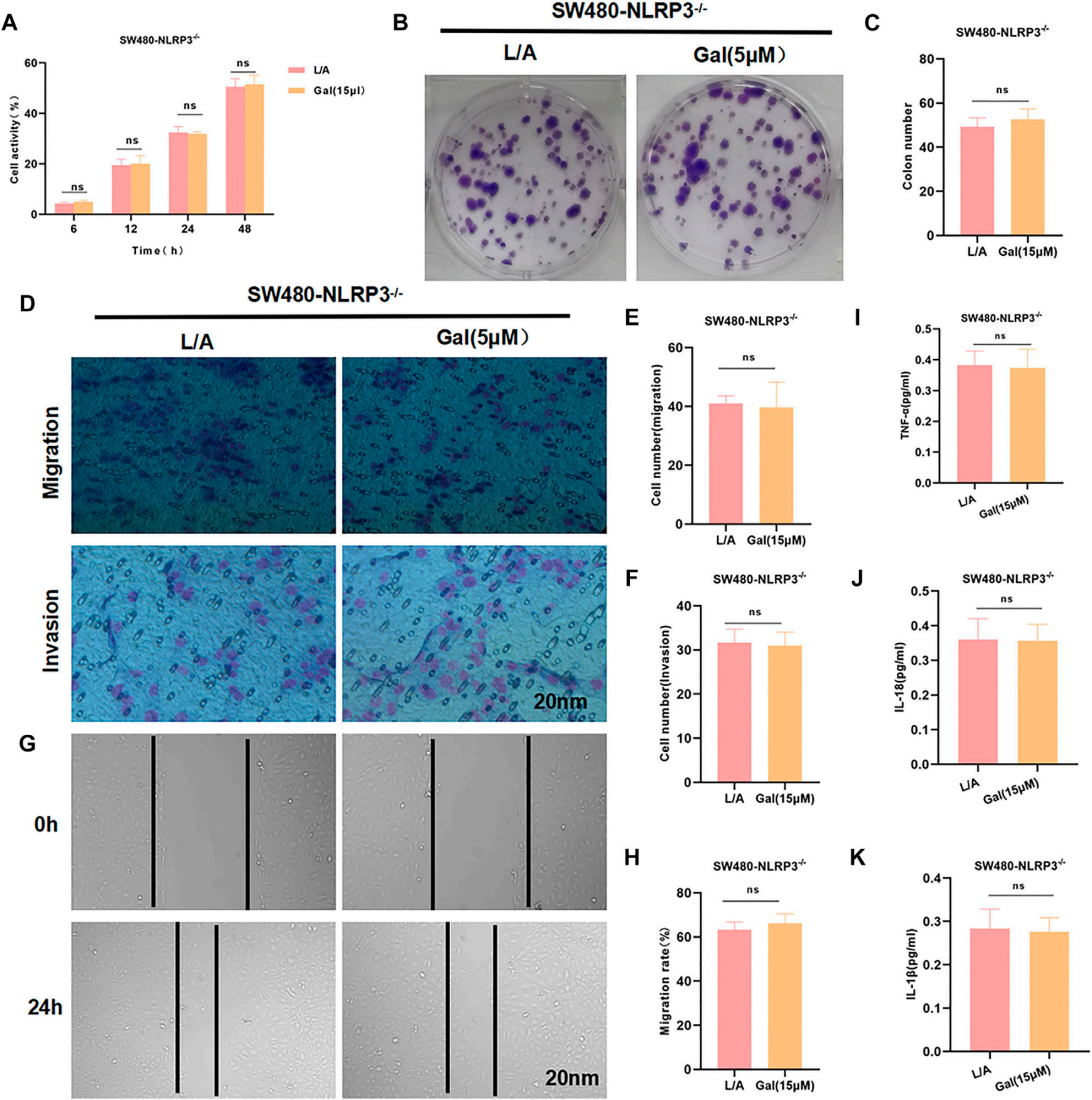
Galloflavin cannot further inhibit the malignant behavior of SW480-NLRP3^−/−^ cells. **(A–H)**: Gal cannot further inhibit cell viability, colony formation, migration, or invasion ability in SW480-NLRP3^−/−^ cells. There was no significant difference, ^ns^ *p* >0.05. **(I–K)**: The detection of inflammatory factors revealed that Gal had no significant effect on the expression of IL-6, TNF-α, or IL-1β in SW480-NLRP3^−/−^ cells. There was no difference between the groups, ^ns^ *p* >0.05.

To investigate whether Gal acted through NLRP3, SW480-NLRP3^−/−^ cells were intervened with 15 μM Gal in, showing Gal could not further inhibit the malignant behavior in SW480-NLRP3^−/−^ cells. Cell viability, colony formation, migration, or invasion ability was not significantly changed between the groups. Meanwhile, Gal had no effect on the level of inflammatory factors (Figure 5).

### The Targeted-Binding Relationship Between Galloflavin and NLRP3

**FIGURE 6:**
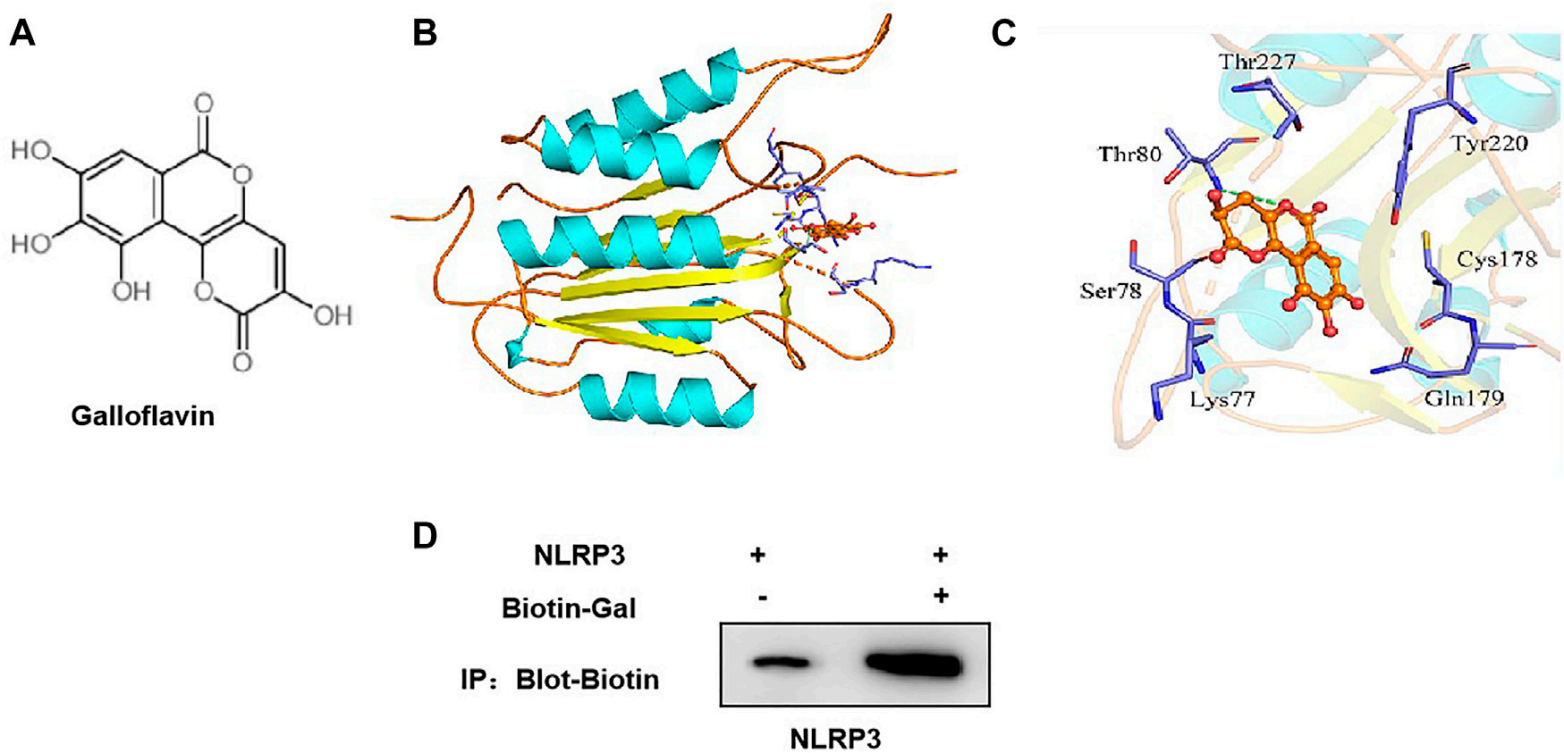
Targeted-binding relationship between galloflavin and NLRP3. **(A)**: Schematic diagram of the molecular structure of galloflavin **(B,C)**: Molecular docking model of galloflavin and NLRP3. **(D)**: Pull-down assay between Biotin–Gal and NLRP3 showed that Gal had a targeted-binding relationship with NLRP3.

The small molecule–protein docking model showed that the amino acid residue Thr80 formed a hydrogen bond with the ligand small molecule Gal, and the amino acid residues Arg79, Tyr220, Cys178, Gln179, Lys77, Ser78, and Thr227 form a hydrophobic bond with the ligand small molecule. Gal could bind to the hydrophobic pocket of NLRP3 and affect the assembly and formation of the NLRP3 inflammasome (Figures 6A–C).

Meanwhile, biotin-labeled Gal was used for protein binding in the pull-down assay. As a result, we found that Gal and NLRP3 had a binding relationship, rather than ASC, which further confirmed the targeted-binding relationship between Gal and NLRP3 (Figure 6D).

### The Effect and Mechanism of Galloflavin on Tumor Growth in Tumor-Bearing Mice

**FIGURE 7:**
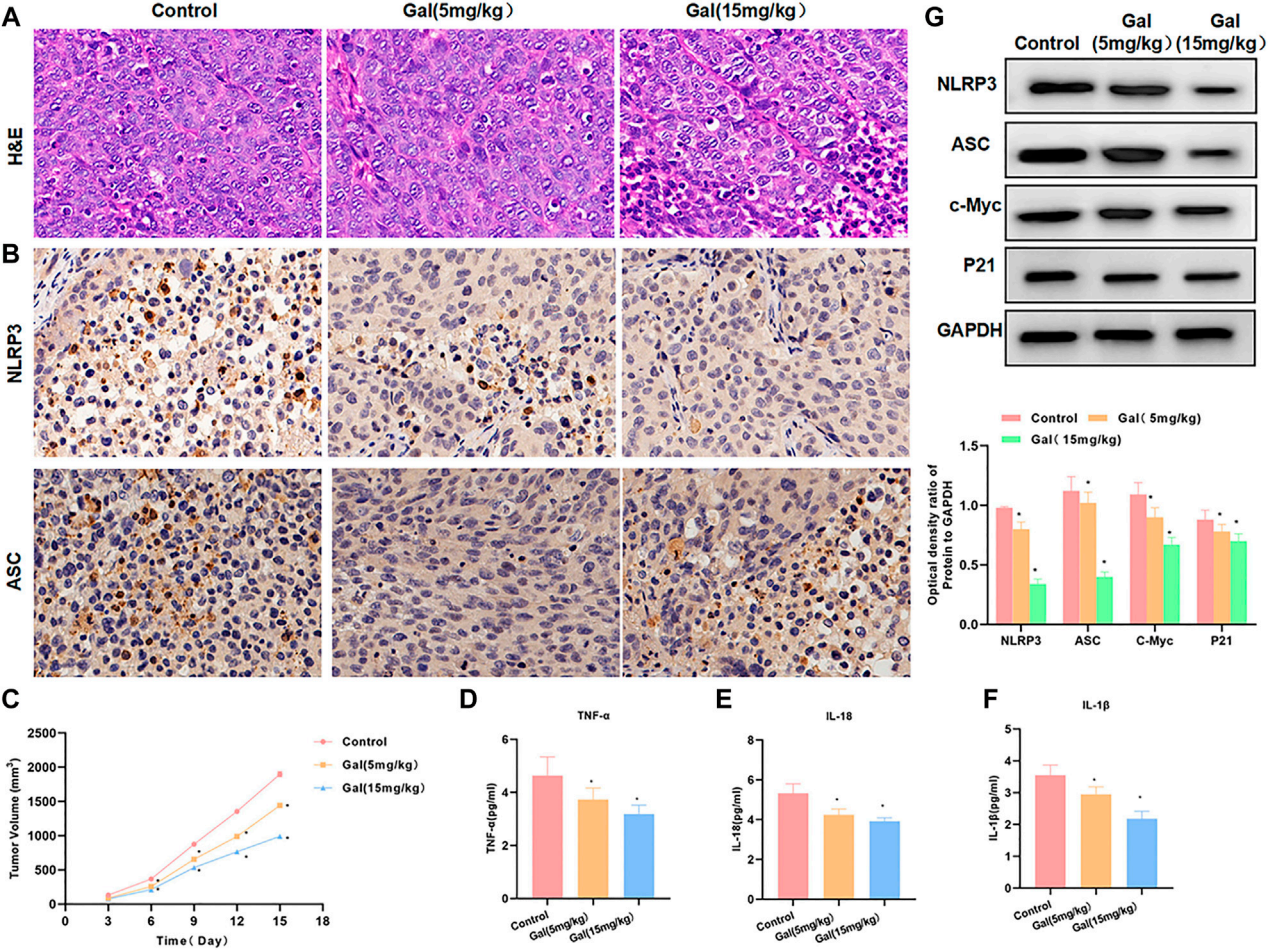
Galloflavin inhibited tumor growth in tumor-bearing mice. **(A)**: H&E staining revealed that Gal could cause certain injury to the tumor tissue, which was obviously changed compared with the control group. **(B)**: The expression levels of NLRP3 and ASC were relatively high in tumor tissues. Gal could inhibit the expression of NLRP3 and ASC, and the protein level was significantly downregulated. **(C)**: The detection of the tumor growth rate revealed Gal could inhibit tumor growth, which was significantly different from Control. Comparison with the Control group, ^*^ *p* < 0.05. **(D–F)**: Gal inhibited the level of inflammatory factors in the tumor, which was significantly lower than Control and relieved the inflammatory microenvironment. Comparison with the Control group, ^*^ *p* < 0.05. **(G)**: Detection of protein expression showed that Gal could inhibit the expression of NLRP3 and suppressed the levels of c-Myc and P21. Comparison with the Control group, ^*^ *p* < 0.05.

We performed Gal intervention in tumor-bearing mice and found that Gal could significantly inhibit tumor growth. From the tumor growth curve, Gal inhibited tumor growth and tumor volume in a time-dependent pattern, and high-dose Gal had a more obvious inhibitory effect on tumor growth (Figure 7C). H&E staining showed that Gal could cause tumor tissue damage, which was associated with the inhibitory effect of Gal on SW480 growth. IHC also revealed a high expression of NLRP3 and ASC in tumors. Gal could inhibit the levels of NLRP3 and ASC, which was consistent with the results of cell experiments (Figures 7A,B). Protein detection also found that Gal could inhibit the expression of NLRP3 and suppress the expression of c-Myc and P21 (Figure 7G). Gal inhibited the level of inflammatory factors in the tumor, which was significantly lower than Control and relieved the inflammatory microenvironment.(Figures 7D–F).

## Discussion

Inflammation and persistent infection play a critical role in tumorigenesis, tumor development, malignant transformation, invasion, and metastasis (Berraondo et al., 2016). Inflammation induces immune responses including T lymphocytes, B lymphocytes, NK cells, dendritic cells, macrophages, and neutrophils (Man and Kanneganti, 2015). The inflammasome is a newly discovered multi-protein complex in the innate immune system in recent years (Bray et al., 2018). For the basic component of inflammasome, NOD-like receptors (NLRs) function as the receptor protein, apoptosis-associated speck-like protein-containing CARD (ASC) functions as the adaptor protein, and caspase-1 acts as the effector protein (Sharma and Kanneganti, 2016). The research of NLRP3 inflammasome in the occurrence and development of colorectal tumors mainly focuses on its mechanism in colitis-associated colorectal cancer (Zhong et al., 2013). Although inflammation is generally considered as a beneficial response to protect against injury factors and infection, chronic intestinal inflammation such as IBD is considered as a risk factor for colorectal cancer (Franchi et al., 2014; Moossavi et al., 2018). In the DSS-induced colon cancer model, NLRP3 promotes tumorigenesis and tumor growth due to increased inflammation and destruction of the intestinal epithelial barrier (Muñoz-Planillo et al., 2013). There are reports that treatment with NLRP3 inhibitors can relieve colitis under DSS exposure (Daley et al., 2017), which might be caused by the local reduction of pro-inflammatory cytokines, including IL-1β, TNF-α, and IFN-γ (Kozma et al., 1994). The tumor-promoting function of NLRP3 inflammasome is also manifested as macrophages can enhance the invasion and metastasis ability of colorectal cancer through the interaction between the NLRP3 inflammasome and tumor cells (Zhai et al., 2013), which may be one of the mechanisms underlying colorectal cancer liver metastasis. Therefore, NLRP3 inflammasome plays an important role in the occurrence and development of colorectal tumors. Therefore, NLRP3 is also considered as a new type of tumor therapeutic target.

Galloflavin is an inhibitor of LDH-A/B, with certain effects on apoptosis. However, the reports of Gal on tumors are extremely limited. In this study, we first predicted the target of Gal through molecular docking and found that Gal and NLRP3 had a targeted-binding relationship. Gal inhibited the formation of NLRP by binding to the hydrophobic pocket of NLRP3. To explore the role of Gal-NLRP3 in colorectal cancer, we first investigated the role of NLRP3 in the colorectal cancer cell line. As a result, we found that the LPS/ATP-induced inflammatory microenvironment could promote cell viability, colony formation, metastasis, and invasion in SW480 cells, indicating that the inflammatory microenvironment could promote the malignant behavior of colorectal cancer. The overexpression of NLRP3 could exacerbate the inflammatory response under LPS/ATP, thereby promoting the malignant behavior of SW480. Therefore, NLRP3 plays an important role in the microenvironment of colorectal cancer. Of note, NLRP3 also increased the expression of c-Myc and P21, which are important tumor-promoting genes to promote tumor progression (Yin et al., 2017). Moreover, its expression is closely associated with NLRP3, showing that NLRP3 is very likely to play a role through c-Myc and P21. Therefore, we validated that NLRP3 played a tumor-promoting role in colorectal cancer. Gal intervention could significantly inhibit the LPS/ATP-induced malignant behavior, suppress the upregulation of cell viability, inhibit cell migration and invasion, and inhibit colony formation. The phenotypic study showed that Gal could inhibit the metastasis and invasion of colorectal cancer cells in the inflammatory microenvironment. In the study of the mechanism, we found that Gal inhibited the expression of NLRP3 and the level of inflammatory factors, and simultaneously suppress the expression of c-Myc and P21. To clarify the targeted-binding relationship between Gal and NLRP3, Gal was labeled with Biotin. The pull-down assay showed the targeted-binding relationship between Gal and NLRP3 and Gal bound to NLRP3 instead of ASC. SW480-NLRP3^−/−^ cells were further treated with Gal. Consequently, after NLRP3 deletion, Gal could not further inhibit cell malignant behavior, which indirectly proved the regulatory relationship between Gal and NLRP3.

In previous studies, it was also found that inhibition of Gal induced growth inhibition of endometrial cancer cells through LDH (Han et al., 2015). In the study of pancreatic cancer, Gal can also inhibit the proliferation of pancreatic cancer cells and enhance the effect of metformin on pancreatic cancer. These research results are similar to our research, which can support our research results.

In the tumor-bearing mouse model of colorectal cancer, we found that Gal could cause tumor tissue damage and inhibit the expression of NLRP3 and ASC in the tumor, which were consistent with the results in SW480 cells. Gal could also inhibit the level of inflammatory factors in the tumor and decrease the expression of c-Myc and P21. The dynamic monitoring of the tumor volume also found that Gal inhibited tumor growth. Collectively, the results of Gal at the cellular level and the animal level were consistent.

## Conclusion

In this study, we have found that NLRP3 in the inflammatory microenvironment can promote the metastasis and invasion of colorectal cancer cells, which is an important factor in promoting colorectal cancer progression. Galloflavin can inhibit the malignant behavior of colorectal cancer cells in the inflammatory microenvironment by targeting NLRP3, which is an important mechanism underlying the inhibitory effect of galloflavin on colorectal cancer progression. Galloflavin is expected to become a new type of drug for colorectal cancer, which deserves further development.

## Data Availability

The original contributions presented in the study are included in the article/Supplementary Material; further inquiries can be directed to the corresponding authors.

## Abbreviations

ATP, adenosine triphosphate; DAMPs, damage-associated molecular patterns; Gal, galloflavin; H&E, hematoxylin–eosin staining; IHC, immunohistochemistry; LPS, lipopolysaccharide; PAMPs, pathogen-associated molecular patterns; TME, tumor microenvironment.

## References

[B1] Bauer J. Emon M. A. B. Staudacher J. J. Thomas A. L. Zessner-Spitzenberg J. Mancinelli G. (2020). Increased Stiffness of the Tumor Microenvironment in colon Cancer Stimulates Cancer Associated Fibroblast-Mediated Prometastatic Activin A Signaling. Sci. Rep. 10 (10), 50–60. 10.1038/s41598-019-55687-6 31919369 PMC6952350

[B2] Berraondo P. Minute L. Ajona D. Corrales L. Melero I. Pio R. (2016). Innate Immune Mediators in Cancer: between Defense and Resistance. Immunol. Rev. 274 (274), 290–306. 10.1111/imr.12464 27782320

[B3] Bray F. Ferlay J. Soerjomataram I. Siegel R. L. Torre L. A. Jemal A. (2018). Global Cancer Statistics 2018: GLOBOCAN Estimates of Incidence and Mortality Worldwide for 36 Cancers in 185 Countries. CA Cancer J. Clin. 68 (68), 394–424. 10.3322/caac.21492 30207593

[B4] Coll R. C. Robertson A. A. Chae J. J. Higgins S. C. Muñoz-Planillo R. Inserra M. C. (2015). A Small-Molecule Inhibitor of the NLRP3 Inflammasome for the Treatment of Inflammatory Diseases. Nat. Med. 21 (21), 248–255. 10.1038/nm.3806 25686105 PMC4392179

[B5] Daley D. Mani V. R. Mohan N. Akkad N. Pandian G. S. D. B. Savadkar S. (2017). NLRP3 Signaling Drives Macrophage-Induced Adaptive Immune Suppression in Pancreatic Carcinoma. J. Exp. Med. 214 (18), 1711–1724. 10.1084/jem.20161707 28442553 PMC5461004

[B6] Franchi L. Eigenbrod T. Muñoz-Planillo R. Ozkurede U. Kim Y. G. Arindam C. (2014). Cytosolic Double-Stranded RNA Activates the NLRP3 Inflammasome via MAVS-Induced Membrane Permeabilization and K+ Efflux. J. Immunol. 193, 4214–4222. 10.4049/jimmunol.1400582 25225670 PMC4185247

[B8] Guo W. Sun Y. Liu W. Wu X. Guo L. Cai P. (2014). Small Molecule-Driven Mitophagy-Mediated NLRP3 Inflammasome Inhibition Is Responsible for the Prevention of Colitis-Associated Cancer. Autophagy 10 (10), 972–985. 10.4161/auto.28374 24879148 PMC4091180

[B9] Han X. Sheng X. Jones H. M. Jackson A. L. Kilgore J. Stine J. E. (2015). Evaluation of the Anti-tumor Effects of Lactate Dehydrogenase Inhibitor Galloflavin in Endometrial Cancer Cells. J. Hematol. Oncol. 8 (1), 2–4. 10.1186/s13045-014-0097-x 25631326 PMC4316809

[B10] He Q. Xue S. Wa Q. He M. Feng S. Chen Z. (2021). Mining Immune-Related Genes with Prognostic Value in the Tumor Microenvironment of Breast Invasive Ductal Carcinoma. Medicine (Baltimore) 100 (100), e25715. 10.1097/MD.0000000000025715 33907159 PMC8084029

[B11] Jiang H. He H. Chen Y. Huang W. Cheng J. Ye J. (2017). Identification of a Selective and Direct NLRP3 Inhibitor to Treat Inflammatory Disorders. J. Exp. Med. 214 (214), 3219–3238. 10.1084/jem.20171419 29021150 PMC5679172

[B12] Kozma L. Kiss I. Szakáll S. Ember I. (1994). Investigation of C-Myc Oncogene Amplification in Colorectal Cancer. Cancer Lett. 81, 165–169. 10.1016/0304-3835(94)90198-8 8012933

[B13] Man S. M. Kanneganti T. D. (2015). Regulation of Inflammasome Activation. Immunol. Rev. 265 (265), 6–21. 10.1111/imr.12296 25879280 PMC4400844

[B14] Moossavi M. Parsamanesh N. Bahrami A. Atkin S. L. Sahebkar A. (2018). Role of the NLRP3 Inflammasome in Cancer. Mol. Cancer 17 (17), 158. 10.1186/s12943-018-0900-3 30447690 PMC6240225

[B15] Muñoz-Planillo R. Kuffa P. Martínez-Colón G. Smith B. L. Rajendiran T. M. Núñez G. (2013). K⁺ Efflux Is the Common Trigger of NLRP3 Inflammasome Activation by Bacterial Toxins and Particulate Matter. Immunity 38 (38), 1142–1153. 10.1016/j.immuni.2013.05.016 23809161 PMC3730833

[B16] Pellegrini C. Fornai M. Colucci R. Benvenuti L. D’Antongiovanni V. Natale G. (2018). A Comparative Study on the Efficacy of NLRP3 Inflammasome Signaling Inhibitors in a Pre-clinical Model of Bowel Inflammation. Front. Pharmacol. 9 (9), 1405. 10.3389/fphar.2018.01405 30559669 PMC6287041

[B17] Perera A. P. Fernando R. Shinde T. Gundamaraju R. Southam B. Sohal S. S. (2018). MCC950, a Specific Small Molecule Inhibitor of NLRP3 Inflammasome Attenuates Colonic Inflammation in Spontaneous Colitis Mice. Sci. Rep. 8 (8), 8618. 10.1038/s41598-018-26775-w 29872077 PMC5988655

[B18] Rada M. Lazaris A. Kapelanski-Lamoureux A. Mayer T. Z. Metrakos P. (2020). Tumor Microenvironment Conditions that Favor Vessel Co-option in Colorectal Cancer Liver Metastases: A Theoretical Model. Semin. Cancer Biol. (71), 52–64. 10.1016/j.semcancer.2020.09.001 32920126

[B20] Sharma D. Kanneganti T. D. (2016). The Cell Biology of Inflammasomes: Mechanisms of Inflammasome Activation and Regulation. J. Cel Biol 213, 617–629. 10.1083/jcb.201602089 PMC4915194 27325789

[B21] Ural E. E. Toomajian V. Hoque Apu E. Veletic M. Balasingham I. Ashammakhi N. (2021). Visualizing Extracellular Vesicles and Their Function in 3D Tumor Microenvironment Models. Int. J. Mol. Sci. 22 (22), 4784–4794. 10.3390/ijms22094784 33946403 PMC8125158

[B22] Wendt E. H. U. Schoenrogge M. Vollmar B. Zechner D. (2020). Galloflavin Plus Metformin Treatment Impairs Pancreatic Cancer Cells. Anticancer Res. 40, 153–160. 10.21873/anticanres.13936 31892563

[B24] Yin Y. Zhou Z. Liu W. Chang Q. Sun G. Dai Y. (2017). Vascular Endothelial Cells Senescence Is Associated with NOD-like Receptor Family Pyrin Domain-Containing 3 (NLRP3) Inflammasome Activation via Reactive Oxygen Species (ROS)/thioredoxin-interacting Protein (TXNIP) Pathway. Int. J. Biochem. Cel Biol 84 (84), 22–34. 10.1016/j.biocel.2017.01.001 28064010

[B25] Zhai H. Fesler A. Schee K. Fodstad O. Flatmark K. Ju J. (2013). Clinical Significance of Long Intergenic Noncoding RNA-P21 in Colorectal Cancer. Clin. Colorectal Cancer 12 (12), 261–266. 10.1016/j.clcc.2013.06.003 24012455

[B26] Zhong Y. Kinio A. Saleh M. (2013). Functions of NOD-like Receptors in Human Diseases. Front. Immunol. 4 (4), 333. 10.3389/fimmu.2013.00333 24137163 PMC3797414

[B27] Zhou D. Wang J. Wang J. Liu X. (2021). Profiles of Immune Cell Infiltration and Immune-Related Genes in the Tumor Microenvironment of HNSCC with or without HPV Infection. Am. J. Transl Res. 13 (13), 2163–2180. 34017381 PMC8129356

